# Case Report: Treatment of a complex hand disfigurement injury by fully shaped reconstruction of a severed finger combined with ectopic banking

**DOI:** 10.3389/fsurg.2024.1488338

**Published:** 2024-12-05

**Authors:** Zhengfeng Jia, Longbao Shi, Weilu Gao, Han Li, Jianzheng Zhang, Weidong Shi, Jiantao Li, Jianwen Zhao

**Affiliations:** ^1^ Senior Department of Orthopedics, The Fourth Medical Center of Chinese PLA General Hospital, Beijing, China; ^2^ National Clinical Research Center for Orthopedics, Sports Medicine and Rehabilitation, Beijing, China; ^3^ Graduate School of Medical School of Chinese PLA Hospital, Beijing, China; ^4^ PLA Rocket Force Characteristic Medical Center, Beijing, China

**Keywords:** ectopic banking, heterotopic replantation, fully shaped reconstruction, digit salvage, functional reconstruction

## Abstract

One of the primary challenges in hand microsurgical reconstruction lies in addressing severe hand injuries, particularly those involving multiple finger amputations, as autologous replantation might not fully restore hand functionality. In such scenarios, fully shaped reconstruction of a severed finger combined with Ectopic banking could yield superior reconstructive outcomes and enhance the amputated limb's function. This case report presents a unique approach that combines ectopic replantation of an amputated finger with interphalangeal reconstruction methods to restore both the form and function of the hand. A 43-year-old female patient suffered an injury to her left hand, leading to ischaemic amputation of all four fingers. Our treatment strategy involved a blend of allograft reimplantation, interdigital reconstruction, iliac bone grafting, and metacarpophalangeal joint fusion to revive the hand's functionality. A year post-surgery, the toe-to-finger reconstructions of the left thumb and middle finger exhibited excellent survival, although the ring and little fingers were lost. The flap located on the dorsum of the left foot healed seamlessly, with uninterrupted blood flow to the toe tips and no complications. As a result, the patient retained three fingers and regained functional activity. Our study underscores that the synergistic approach of fully shaped reconstruction of a severed finger combined with Ectopic banking not only elevates surgical efficiency but also significantly bolsters hand morphology and function. This case report underscores the significance of the Treatment of a complex hand disfigurement injury by fully shaped reconstruction of a severed finger combined with Ectopic banking in the realm of finger reconstruction, highlighting their transformative potential in restoring hand function and appearance.

## Introduction

Crippling hand injuries are one of the greatest challenges facing hand microsurgeons (1–3), Re-implantation of severed fingers is undoubtedly the best option for restoring function, especially when multiple fingers are involved, and improvements in microsurgical techniques and effective postoperative monitoring have led to an increasing survival rate of reimplantation (4, 5). However, as medical technology continues to advance, the challenge is not only to restore blood circulation to the fingers but also to better restore hand function (6).

Therefore, in the treatment of crippling hand injuries, it is of utmost importance to restore a working finger and to restore normal joint movement and motor stability, as well as sensory sensitivity in the fingers (7). With advances in microsurgery and medicine, a variety of finger reconstruction techniques have been developed and ectopic fostering techniques have matured; however, each technique has its limitations, including simple toe grafts and finger reconstruction are aesthetically unpleasant (8); Prosthetic fingers can physically replace fingers, but not sensorimotor functions (9, 10); Connected bilateral second toes for long finger reconstruction are associated with poor function and appearance (11). In this trend, the concept of holomorphic reconstruction was born (3, 12), finger reconstruction has been widely carried out at home and abroad.

Fully shaped reconstruction of a severed finger combined with ectopic banking is an innovative technique for the repair of residual hand injuries that can compensate for the limitations of previous approaches. Although heterotopic fostering composite repair has been in use for many years, few reports have focused on the clinical outcomes of ectopic banking composite repair in disfiguring hand injuries. Here, we report a complex case of disfiguring hand injury treated with heterotopic fostering of a severed finger and reconstructed fingers from a pedicle, and evaluate the existing literature.

### Case report

The patient signed a paper version of the informed consent form, and it was approved by the Ethics Committee of the PLA General Hospital.

### General information

**Figure 1:**
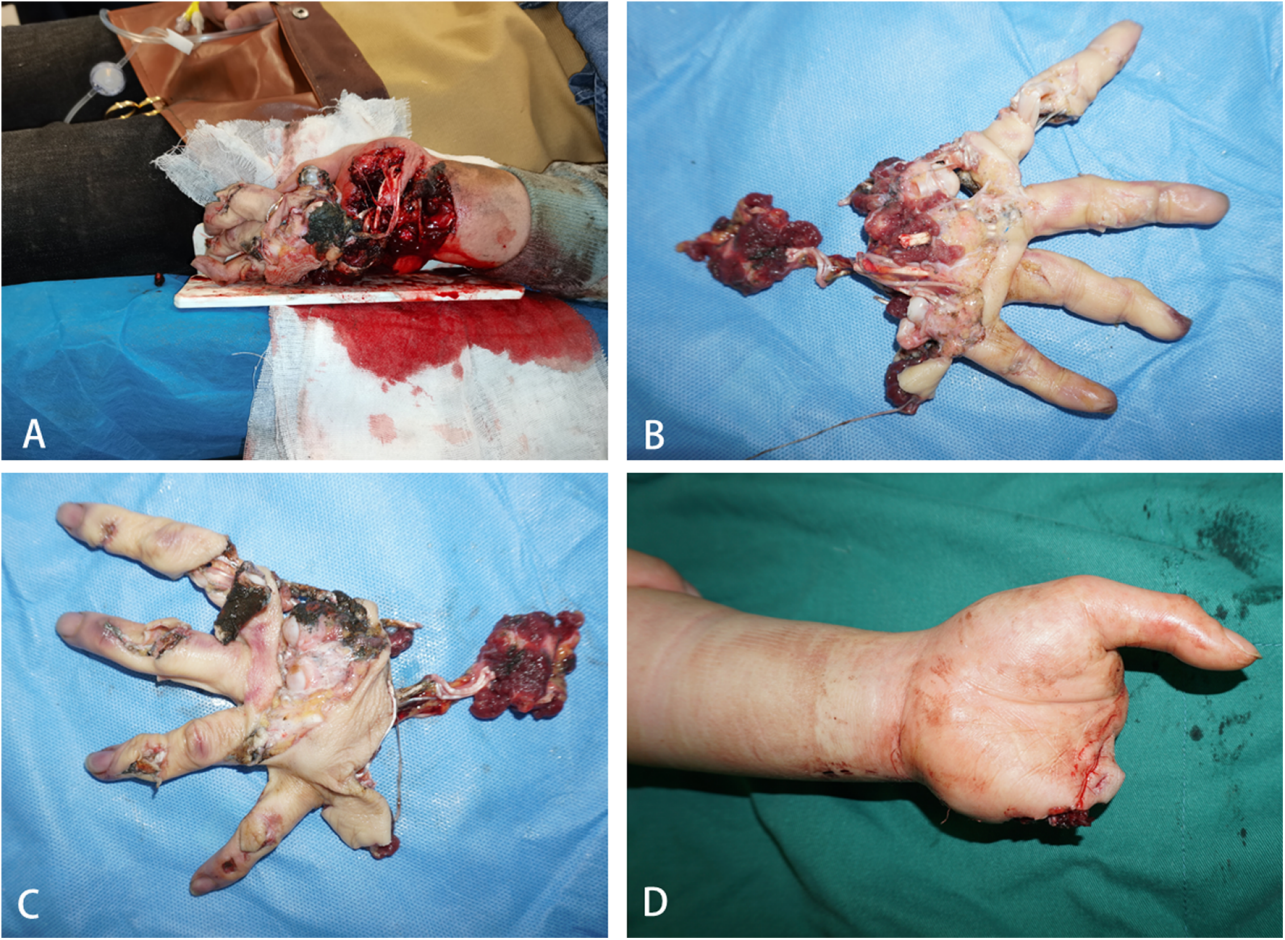
**(A**–**D)** on admission, examination revealed severe damage to the left index, middle, ring, and little fingers. The distal ends showed no blood circulation, and there was extensive damage beyond the mid-palm level, accompanied by multiple wounds and exposure of tendons and metacarpal bones.

A male patient, 43 years old, was admitted to the hospital with a “Meat grinder injured left hand for three hours.” She was admitted to the hospital on an emergency basis after three hours of severe destruction of her left hand due to the inadvertent operation of a meat grinder at home. x-ray examination showed multiple metacarpal and phalangeal fractures of the left hand and poor alignment of the carpometacarpal and metacarpophalangeal joints (Figure 1).

### Treatments

**Figure 2:**
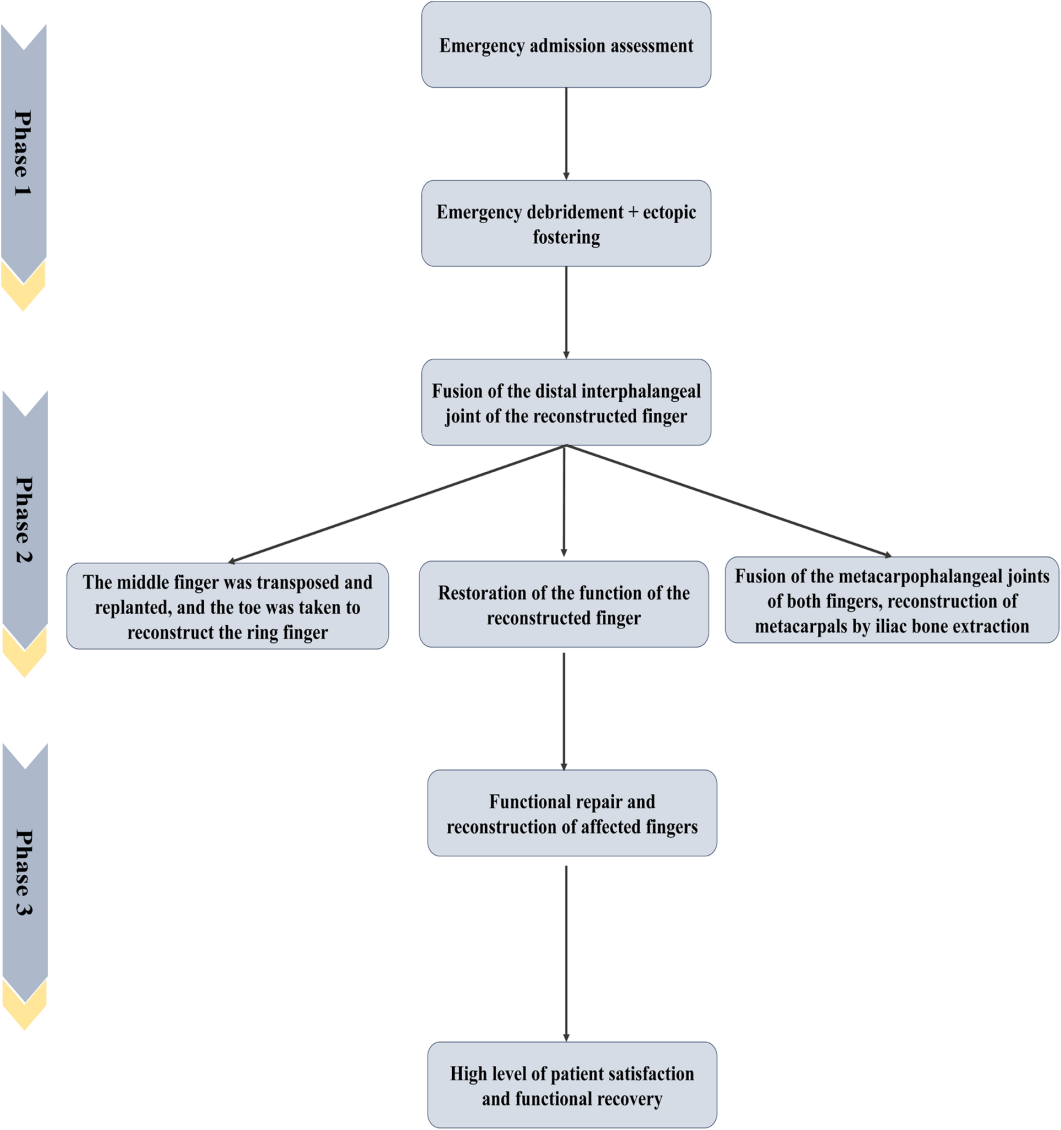
Disposal procedure and surgical plan.

Given the complexity of the patient's injury and the severe damage to the palm, primary replantation is not suitable. After thorough discussion, our surgical team has decided to adopt a treatment plan involving the ectopic replantation of the severed finger to the foot. This will be followed by a secondary procedure to repair the bone and flap in the palm and simultaneously transplant the replanted finger back to the hand (Figure 2).

### Treatment process and results

#### Phase 1: ectopic fostering

After completing the relevant preoperative preparations, we carefully assessed the patient's severed fingers and the condition of the remaining blood vessels. For the primary procedure, we opted for debridement and heterotopic finger grafting, placing each of the four fingers on the dorsum of the feet. Under general anesthesia, we thoroughly debrided the wound on the left hand and skillfully utilized the dorsal pedis vessels (particularly the dorsal pedal artery) as anastomosis sites to graft the severed fingers onto the dorsum of the feet. Postoperatively, we followed the standard care protocol for finger reattachment (Figure 2). After one month of meticulous care, the blood supply to the middle finger grafted on the left foot was satisfactory, while the other three fingers underwent necrosis. The patient was able to fully bear weight and walk ten days after the surgery and was successfully discharged.

#### Phase 2: replantation of the middle finger and reconstruction of the ring finger using a Toe

**Figure 3:**
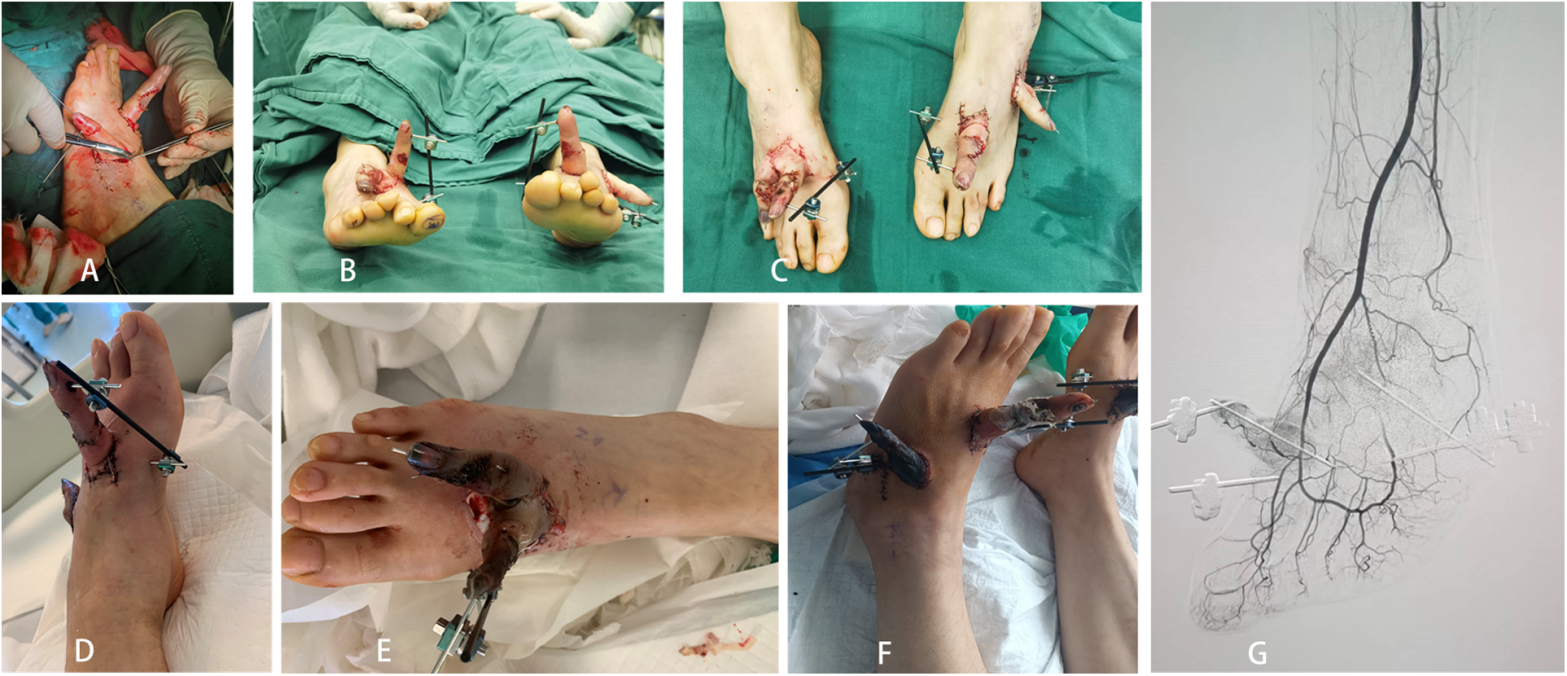
**(A**–**C)** One-phase surgery was performed for debridement and ectopic fostering of the severed fingers, with each of the four fingers being fostered in both feet; **(D**–**F)** after a month of careful care, the middle finger of the left foot was foster was in good hematological condition, while the remaining three fingers were necrotic; **(G)** post-operative DSA examination.

Six weeks post-trauma, we plan to perform the second stage of surgery. Considering that only the middle finger has survived, a hand with just the thumb and middle finger would not have good grasping functionality. Additionally, if we proceed with a staged approach, where the fostered finger transplant is done first followed by reconstruction, the blood supply during the fostering phase will have already established connections with the first dorsal metatarsal artery, thereby increasing the risk of staged surgery. Therefore, we innovatively decided to perform the foster finger transplant and finger reconstruction simultaneously. This includes the relocation and replantation of the middle finger, as well as the reconstruction of the index finger using a toe from the foot. The procedure was performed as described below (Figure 3).

**Figure 4:**
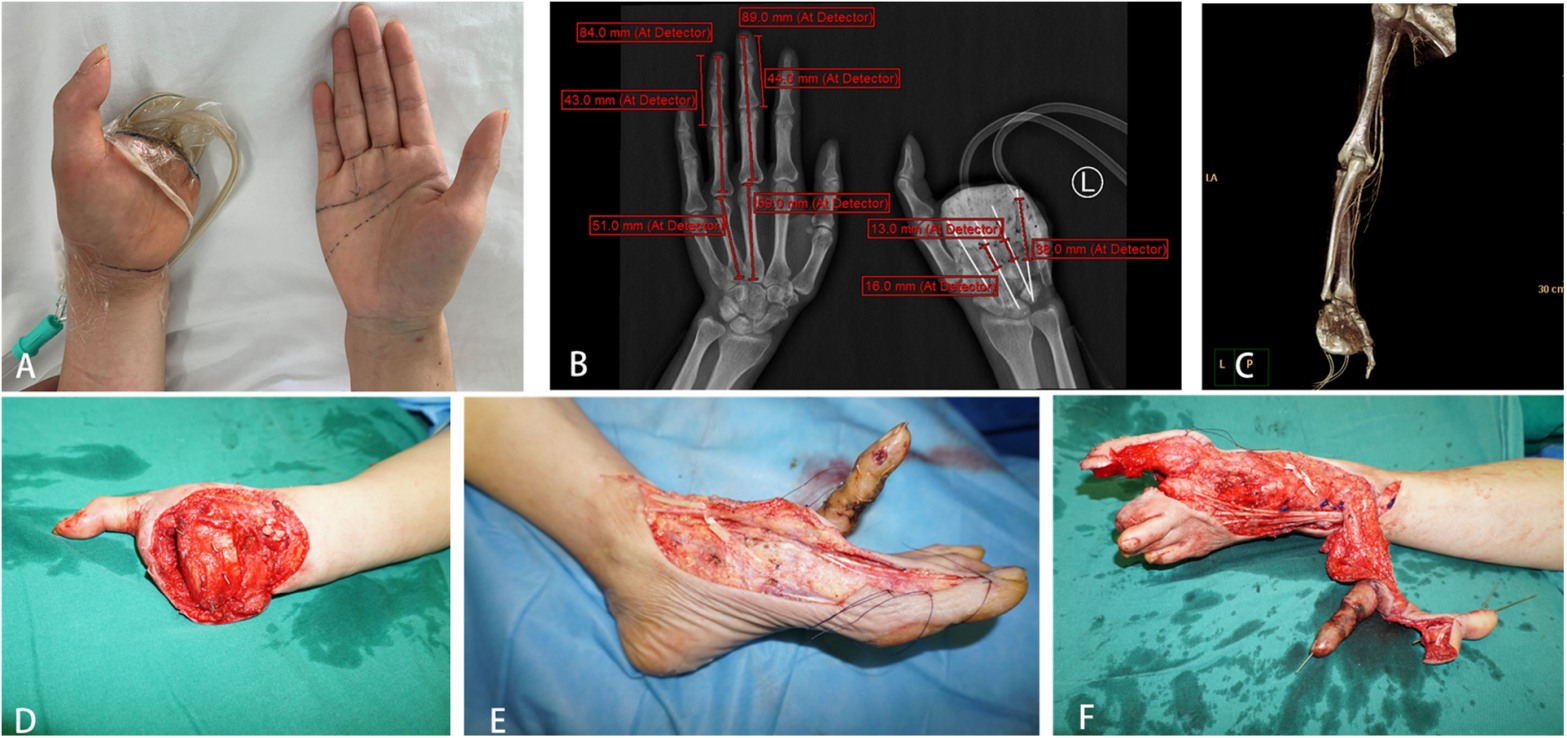
**(A**–**C)** precise pre-operative planning; **(D**–**F)** replantation of the middle finger and reconstruction of the ring finger by removal of the toe.

The palmar defect was reconstructed using a 3.5 × 4 cm iliac bone graft. The reconstructed index finger was formed by combining skin from the great toe, the interphalangeal joint of the second toe, and part of the iliac bone, along with the fostered finger. Additionally, a composite tissue flap from the dorsal foot, including a long pedicle (comprising the dorsal foot artery and its accompanying veins, as well as the great saphenous vein), was meticulously anastomosed to the deep branch of the radial artery and the cephalic vein. This approach allowed us to reconstruct the patient's palmar defect and restore the morphology of the index and middle fingers simultaneously. The donor site on the foot was repaired using a contralateral anterolateral thigh flap. Due to the prolonged surgical time and the extensive tendon defects, the motor function of the reconstructed index and middle fingers was not restored during this procedure. Three weeks post-surgery, the transplanted fostered finger and the reconstructed index finger had survived well. The patient then began passive movement of the thumb, gradually progressing to active movement over the next six weeks (Figure 4).

#### Phase 3: hand function reconstruction

In the subsequent treatment, we focused on the functional reconstruction of the patient's left hand. Through multiple surgeries, the patient underwent exploration and allograft tendon transplantation for the extensor and flexor tendons of the left hand's index and middle fingers. Additionally, we performed thinning of the left foot flap, exposed phalanx removal, debridement, and suturing. We successfully restored partial function to the patient's left hand. After seven months of treatment and rehabilitation, the survival of the reconstructed index and middle fingers (toe-to-hand transfer) was satisfactory. Although there were limitations in movement, the overall recovery outcome was considered satisfactory (Appendix A1).

### Treatment outcomes, follow-up, and prognosis

Throughout the treatment, we consistently monitored the patient's feedback and satisfaction (13). Monthly follow-ups were conducted according to the rehabilitation plan, documenting complications, limb mobility, patient-reported satisfaction, Visual Analog Scale (VAS) scores (14), Short Form-36 (SF-36) scores (15, 16), and imaging data. One year after the first-stage surgery, VAS scores indicated a significant improvement, with the patient's pain level decreasing from 8 before surgery to 2 postoperatively. This suggests a marked reduction in pain and discomfort. Additionally, the SF-36 health survey results demonstrated high scores in physical functioning, mental health, and overall health status (Table 1). These positive outcomes not only validate the effectiveness of our treatment plan but also provide valuable experience and reference for future cases.

**Table 1:** Short form-36 health survey questionnaire results.

SF-36 Scales^a^	Scores
Physical functioning	75
Physical role	50
Pain index	40
General health	85
Vitality	65
Social function	60.5
Emotional role	77
Mental health index	80

SF-36, Short Form-36 Health Survey.

^a^ All the SF-36 scales are based on a scale of 0–100. For all scales, a higher score represents better hand function. For the pain scale, a higher score indicates less pain.

In conclusion, the complex hand injury case, managed with dislocated finger transplantation and complete reconstruction, achieved satisfactory results. The successful application of this innovative technique highlights our expertise in hand surgery and offers new insights and directions for treating similar cases.

## Discussion

The main difference between this case report and previous cases is the first successful combined application of ectopic parasitosis, finger reconstruction, and flap grafting for hand reconstruction. This innovative technique has achieved a breakthrough in the field of hand surgery, providing a new way of thinking about the treatment of complex hand injuries and achieving satisfactory clinical results and patient satisfaction.

Complex disfiguring injuries to the hand have been a great challenge in hand surgery (2). Conventional *in situ* reimplantation surgery is not optimal in some cases due to the need for significant shortening of the severed finger and the low success rate. In this case, the challenges we faced included not only the complexity of the surgery itself, but also how to apply a combination of ectopic banking, toe reconstruction, and fully shaped reconstruction to achieve optimal restoration of hand function.

By analyzing the existing literature and clinical judgment, we chose ectopic banking as the initial management, followed by the reconstruction of finger and iliac bone harvesting reconstruction, combined with foot-free flap repair. This three-stage repair plan is relatively rare in the existing literature but has a solid theoretical and practical basis. The ultimate goal of surgical reimplantation is not only to achieve successful revascularisation but more importantly the restoration of hand function and patient satisfaction (17–19). Heterotopic replantation offers significant advantages in patients with extensive soft tissue loss or severe contamination requiring radical debridement. However, there is no consensus on the indications for surgery, the ideal location and duration of ectopic banking, the approach and timing of soft tissue management, and the management of complex microvascular secondary reimplantation (20, 21). In reported cases of primary ectopic banking, the reimplanted portion is usually transferred to the correct position within a few weeks after the initial ectopic banking, an approach that is effective in restoring hand function. Bakhach et al (22) demonstrated the effectiveness of ectopic banking in hand reconstruction by performing ectopic banking in four patients, after stump reconstruction, the ingers were microsurgically transplanted to the hand, and functional pinching was eventually restored. In addition, alloplastic reconstruction achieves the goal of “how much is missing, how much is being replaced”, which makes the repair and reconstruction of finger defects precise, but does not emphasize the control of damage to the donor area (23). Holomorphic reconstruction, proposed by Wang Zengtao in 2011, emphasizes the design and assembly of a functionally and cosmetically near-normal finger according to the structure and shape of the healthy finger, while preserving the length and most of the function and shape of the toes (24). This technique allows for more precise repair and reconstruction of finger defects. It is currently the optimal solution for finger reconstruction.

Fully shaped reconstruction of a severed finger combined with Ectopic banking presented in this case is an innovative surgical approach that emphasizes the design and assembly of a finger that is functionally and cosmetically close to normal based on the structure and shape of a healthy finger while preserving as much as possible the length, function, and morphology of the pedicle. Compared with conventional finger replantation techniques, ectopic parasitism combined with holomorphic reconstruction of severed fingers is not only more precise in terms of surgical design, but also better able to preserve the function and appearance of the finger. It has been mentioned in the literature that although the ectopic fostering technique has been used for many years, there are relatively few reports of combining it with alloplastic reconstruction for the management of complex hand disfigurement. This paper demonstrates the effectiveness of this innovative technique through the successful treatment of complex hand disfigurement injuries. The clinical significance of this technique lies in the fact that it provides a new treatment option for complex hand disfigurement injuries that are difficult to treat by traditional surgical methods.

Of course, the technique of ectopic banking has matured and the technique of alloplastic reconstruction is increasingly being applied. The innovation of this case lies in the combination of ectopic banking and fully shaped reconstruction to repair the hand injury, and the difficulty involves simultaneous grafting of blood vessels for reconstruction and the need for simultaneous surgeries, which makes the surgery difficult and requires a very high level of demands on the surgical team. To the best of our knowledge, this is the first case in the world where this combined technique has been successfully applied for hand reconstruction. After overcoming all stages of surgical difficulty, we achieved remarkable clinical success, providing valuable experience and new ideas for the treatment of similar complex hand injuries.

Fully shaped reconstruction of a severed finger combined with ectopic banking is a difficult multi-stage microvascular procedure, while combined reconstruction complicates the difficulty of the procedure and should only be considered for very valuable sites (25). The risk of potentially serious complications (e.g., sepsis and hemorrhage) exists when salvaging a crippled hand trauma by allografting. Therefore, this technique should only be considered if performed by a high-level reconstruction team in a microsurgical setting. In addition, the success of ectopic banking relies on meticulous postoperative management and a rigorous rehabilitation program to ensure the survival and functional recovery of the transplanted tissue.

With the development of technology and more clinical experience, the combined application of fully shaped reconstruction of a severed finger with ectopic banking is expected to be further optimized to improve the success rate of hand reconstruction surgery and the quality of life of patients. Future studies should focus on the refinement of surgical indications, management of postoperative complications, and assessment of long-term functional recovery to provide more comprehensive treatment options in the field of hand surgery.

## Conclusion

Fully shaped reconstruction of a severed finger combined with ectopic banking is a difficult multistage microvascular procedure. This case report demonstrates the value of ectopic parasitism with pedicle reconstructive fingers in finger reconstruction. It not only validates the feasibility and effectiveness of this innovative surgical approach but also provides a valuable reference for the treatment of similar cases in the future.

## Data Availability

The datasets presented in this article are available upon reasonable request. Requests to access the datasets should be directed to zhengfengjia301@163.com.

## Appendix

A1. (A) Reconstructive preoperative x-rays; (B–D) functional reconstruction of the left demonstrative and little finger flexion under brachial plexus anesthesia with internal fixation removal; (E,F) postoperative x-rays and dressing changes show good reconstruction.

## References

[1] Wolfe VM Wang AA. Replantation of the upper extremity. J Am Acad Orthop Surg. (2015) 23(6):373–81. 10.5435/JAAOS-D-14-00039 26001429

[2] Bueno A Nevado-Sanchez E Collazo C De la Fuente-Anuncibay R González-Bernal J. Functional outcomes in upper limb replantation-a systematic review. J Clin Med. (2024) 13(5). 10.3390/jcm13051289 PMC10931822 38592128

[3] Hou C Zhang A Yu X Han H. Reconstruction of fully shaped fingers using a free great toe nail flap combined with a second toe tissue flap. Int Wound J. (2022) 19(6):1389–96. 10.1111/iwj.13732 35611596 PMC9493232

[4] Godina M Bajec J Baraga A. Salvage of the mutilated upper extremity with temporary ectopic implantation of the undamaged part. Plast Reconstr Surg. (1986) 78(3):295–9. 10.1097/00006534-198609000-00003 3737753

[5] Cavadas PC Landin L Navarro-Monzones A Soler-Nomdedeu S. Salvage of impending replant failure by temporary ectopic replantation: a case report. J Hand Surg Am. (2006) 31(3):463–7. 10.1016/j.jhsa.2005.12.016 16516743

[6] Ono S Chung KC. Efficiency in digital and hand replantation. Clin Plast Surg. (2019) 46(3):359–70. 10.1016/j.cps.2019.03.002 31103081 PMC6527348

[7] Pet MA Ko JH Vedder NB. Reconstruction of the traumatized thumb. Plast Reconstr Surg. (2014) 134(6):1235–45. 10.1097/PRS.0000000000000716 25255109

[8] Forte AJ Maita KC Torres-Guzman RA Avila FR Safa B Buncke G Great toe transplantation. Semin Plast Surg. (2022) 36(4):243–52. 10.1055/s-0042-1758689 36561428 PMC9762995

[9] Elbeshbeshy B Paksima N. Post-traumatic thumb reconstruction. Bull Hosp Jt Dis. (2001) 60(3-4):130–3. 12102399

[10] Pet MA Morrison SD Mack JS Sears ED Wright T Lussiez AD Comparison of patient-reported outcomes after traumatic upper extremity amputation: replantation versus prosthetic rehabilitation. Injury. (2016) 47(12):2783–8. 10.1016/j.injury.2016.10.004 28029356

[11] Wei FC Chen HC Chuang CC Noordhoff MS. Reconstruction of the thumb with a trimmed-toe transfer technique. Plast Reconstr Surg. (1988) 82(3):506–15. 10.1097/00006534-198809000-00025 3406184

[12] de Oña I R Villanueva A G de Oya A S. An alternative thumb reconstruction by double microsurgical transfer from the great and second toe for a carpometacarpal amputation. J Hand Surg Am. (2018) 43(10):955.e1–.e9. 10.1016/j.jhsa.2018.03.022 29705012

[13] Maraccini AM Slonim AD. Prioritizing the assessment of patient and family experience in the evaluation of healthcare quality. Pediatr Crit Care Med. (2020) 21(10):905–6. 10.1097/PCC.0000000000002551 33009303

[14] Collins SL Moore RA McQuay HJ. The visual analogue pain intensity scale: what is moderate pain in millimetres? Pain. (1997) 72(1-2):95–7. 10.1016/S0304-3959(97)00005-5 9272792

[15] Contopoulos-Ioannidis DG Karvouni A Kouri I Ioannidis JP. Reporting and interpretation of SF-36 outcomes in randomised trials: systematic review. BMJ. (2009) 338:a3006. 10.1136/bmj.a3006 19139138 PMC2628302

[16] Reed PJ Moore DD. SF-36 as a predictor of health states. Value Health. (2000) 3(3):202–7. 10.1046/j.1524-4733.2000.33005.x 16464184

[17] Barbato B Salsac AV. Finger and thumb replantation: from biomechanics to practical surgical applications. Hand Surg Rehabil. (2020) 39(2):77–91. 10.1016/j.hansur.2019.10.198 31837487

[18] Barbary S Dap F Dautel G. Finger replantation: surgical technique and indications. Chir Main. (2013) 32(6):363–72. 10.1016/j.main.2013.04.012 24075814

[19] Tuzun HY Turkkan S Arsenishvili A Kurklu M. A new technique for metacarpophalangeal joint replantation after four-finger amputation. Hand Surg Rehabil. (2020) 39(3):235–7. 10.1016/j.hansur.2020.01.007 32088425

[20] Higgins JP. Ectopic banking of amputated parts: a clinical review. J Hand Surg Am. (2011) 36(11):1868–76. 10.1016/j.jhsa.2011.09.003 22036286

[21] Yoshida S Koshima I Narushima M Nagamatsu S Yokota K Yamashita S Usefulness of ectopic implantation in multiple finger amputation injury. Clin Case Rep. (2019) 7(3):546–9. 10.1002/ccr3.2040 30899491 PMC6406161

[22] Bakhach J Katrana F Panconi B Baudet J Guimberteau JC. Temporary ectopic digital implantation: a clinical series of eight digits. J Hand Surg Eur. (2008) 33(6):717–22. 10.1177/1753193408091429 18694920

[23] Yin Y Tao X Li Y Bao B Ying Y Bao T Cosmetic and functional results of a newly reconstructed thumb by combining the phalanx of second toe and the great toenail flap transplantation. J Orthop Surg Res. (2020) 15(1):458. 10.1186/s13018-020-01986-y 33023628 PMC7542354

[24] Yu F Xiao F Peng G Lin G Wang W Xie C Repair of distal finger soft-tissue defects with free fibular great toe neurovascular flaps. BMC Musculoskelet Disord. (2024) 25(1):479. 10.1186/s12891-024-07563-2 38890706 PMC11184890

[25] Retrouvey H Franks A Dunn T Novoa K Ipaktchi K Lauder A. Management of self-inflicted nonaccidental amputations of the upper extremity: systematic review. J Hand Surg Am. (2023) 48(10):993–1002. 10.1016/j.jhsa.2023.06.011 37589622

